# Improving power in genetic-association studies via wavelet transformation

**DOI:** 10.1186/1471-2156-10-53

**Published:** 2009-09-11

**Authors:** Renfang Jiang, Jianping Dong, Yilin Dai

**Affiliations:** 1 Department of Mathematical Sciences, Michigan Technological University, USA

## Abstract

**Background:**

A key to increasing the power of multilocus association tests is to reduce the number of degrees of freedom by suppressing noise from data. One of the difficulties is to decide how much noise to suppress. An often overlooked problem is that commonly used association tests based on genotype data cannot utilize the genetic information contained in spatial ordering of SNPs (see proof in the Appendix), which may prevent them from achieving higher power.

**Results:**

We develop a score test based on wavelet transform with empirical Bayesian thresholding. Extensive simulation studies are carried out under various LD structures as well as using HapMap data from many different chromosomes for both qualitative and quantitative traits. Simulation results show that the proposed test automatically adjusts the level of noise suppression according to LD structures, and it is able to consistently achieve higher or similar powers than many commonly used association tests including the principle component regression method (PCReg).

**Conclusion:**

The wavelet-based score test automatically suppresses the right amount of noise and uses the information contained in spatial ordering of SNPs to achieve higher power.

## Background

In a genome-wide association study (GWAS), if a SNP has a strong LD with a disease locus, single marker methods should have more power than multiple marker methods. However, if several SNPs have moderate associations with disease genes, multiple marker methods (such as Hotelling's *T*^2 ^test [1-4] or multiple logistic regression) can provide higher power [5]. One of the problems of multiple marker methods is their large number of degrees of freedom, which in turn may lead to low power. Therefore, reducing the number of degrees of freedom becomes a key issue in gaining power for a multilocus method. For example, in haplotype association studies, tests based on haplotype sharing [6,7] have fewer degrees of freedom and higher power than tests based on haplotype frequencies. Another common approach to reducing the number of degrees of freedom is PCReg [8,9]. Projections of genotype data onto a few principal component directions of the variance-covariance matrix can often capture a majority of genotypic variances, and have fewer degrees of freedom. Tests based on projected genotype data can potentially have higher power. A weighted score test based on the Fourier transform [10] was also introduced to reduce the number of degrees of freedom. The basic idea behind this test is to transform genotype data into Fourier coefficients, and form the test based on those Fourier coefficients. Noise reduction is done by putting high weights on low frequency components, and low weights on high frequency components. The rationale behind this approach is a belief that assuming the signal function belongs to a certain smoothness class, high frequency components are mostly noise and therefore should be suppressed. This method works well if genotypic variation is across all SNPs. Under the common disease and rare variant hypothesis, a collapsing method and a combined multivariate and collapsing method were proposed [11], in which the genotypes of rare variants are collapsed to reduce the number of degrees of freedom and to increase power.

Reducing the number of degrees of freedom in genetic studies is similar to suppressing noisy data; the difficulty is to know how much of the data should be suppressed. A good test should adapt to LD structures, which means it can automatically decide the amount of noise to be suppressed. In a window of SNPs, if most of the SNPs are not associated with the disease, then most genotypic variations are noise, which should all be suppressed. On the other hand, if most of the SNPs are in fact associated with the disease, then these genotypic variations are true signals and therefore should be kept. An ideal noise suppression (de-noising) process should automatically choose an optimal suppression level to maximize the signal to noise ratio. We propose a novel test, which is able to automatically choose an optimal suppression level.

Many tests based on genotype data, for example, Hotelling's *T*^2 ^test [1-4], logistic regression test and PCReg [8], do not take the spatial order of the SNPs on a chromosome into consideration (see proof in the Appendix). As a result, interchanging the relative positions of two SNPs does not have an effect on the tests (see proof in the Appendix). The test we proposed in this paper takes the order of the SNPs on a chromosome into consideration, and it gains power by doing so. Tests based on haplotype data also consider the order of SNPs on a chromosome. For example, the ordering of SNPs is essential for tests based on haplotype similarity (the longest continuous interval of matching alleles between haplotypes). If the relative order of SNPs is changed, the shared length between two haplotypes will be reduced and the disease association cannot be detected. If we assume affected individuals share some ancestral haplotypes, the relative positions of SNPs on a chromosome contain genetic information associated with a disease, and ignoring this information may reduce the power of genetic association tests. The proposed test is a compromise between these genotype-based and haplotype-based tests in the sense that the ordering of SNPs is considered but ambiguous haplotypes do not need to be inferred.

## Results and Discussion

### Wavelet transform

Wavelet transformation is a method for decomposing data into different frequency components. It is an effective noise suppressing (de-noising) method, and it is designed to deal with choppy signals. Because of its adaptability to jumps, small wiggles, and other unusual features in the target function, it has become an important tool to replace the Fourier transform in many practical situations. The introduction of the wavelet method in statistics began more than a decade ago [12]. Since then it has been applied to many areas of statistics including nonparametric regression, time series analysis, nonparametric density estimation, and contingency table cell probability estimation [13].

Dilation and translation of a pair of father and mother wavelets {*φ*, *ψ*} generates an orthonormal wavelet basis for the space of square-integrable functions. A square-integrable function *f*(*x*) can be written as a wavelet series:



where *c*_*i*, *j *_are the wavelet coefficients and *φ*_*i*, *j *_and *ψ*_*i*, *j *_are the wavelet basis functions. A discrete wavelet transform changes discrete data to wavelet coefficients. There are many choices for the father and mother wavelets, and they are chosen to give the wavelet transformation desired properties: to suppress noise more effectively; to be easily adapted to dense or sparse signals, and to deal with unsmooth functions with unusual features. Noise suppression is achieved by removing terms from the above summation (letting some wavelet coefficients be zero), while the main features of the target function can still be kept. Computing discrete wavelet coefficients is faster than computing Fourier coefficients. The time required for calculating discrete wavelet coefficients of *n *data points is *O*(*n*), and that for Fourier coefficients is *O*(*n *log *n*).

There are three steps in a wavelet transformation. Step one is to transform genotype scores to wavelet coefficients. Step two decides which wavelet coefficients to be removed or shrunk, and this process is called thresholding. Step three transforms the modified wavelet coefficients back to modified genotype scores. The key to reducing the number of degrees of freedom and increasing the power lies in step two: thresholding. A wavelet coefficient is kept if it is greater than a high threshold, it is dropped if it is smaller than a low threshold, and it is shrunk if it lies in between the two thresholds. For a data set, one can choose a single threshold for all wavelet coefficients, or choose multiple thresholds to handle wavelet coefficients on different resolution levels and with different frequencies. One can also choose thresholds manually. But this will not work for a GWAS. The scale of a GWAS calls for a data-dependent choice of thresholds. The simplest data-dependent threshold is a universal single threshold [12], which is given by , where *σ *is the standard deviation of noise and *n *is the sample size. It is generally believed that at high resolution levels the wavelet coefficients of the signal are more sparse than those in the low resolution levels, therefore, a level-dependent choice of thresholds is better than the universal threshold. A false discovery rate (FDR) method has been used in choosing level-dependent thresholds [14]. While it works well for sparse signals, it does not adapt very well to dense signals. Many other thresholding methods have been proposed, and this is still an active research area.

We chose the empirical Bayesian thresholding [15]. The advantages of this approach include its superior adaptability to sparse and dense signals. It is a data-dependent, automatic procedure. Let *C*_*i *_= *μ*_*i *_+ ϵ_*i *_be the sample wavelet coefficients at a certain resolution level, where ϵ_*i *_represents noise. Suppose the signal *μ*_*i *_has a prior distribution (1 - *w*)*δ*_0 _+ *wγ*, where *δ*_0 _is an atom probability at zero and *γ *is a unimodal symmetric density. Let (*c, w*) be the median of the posterior distribution of *μ*, given *C *= *c*. There exists *t*(*w*) such that (*c*, *w*) = 0 if and only if |*c*| ≤ *t*(*w*). If  is the marginal maximum likelihood estimator of *w*, then the empirical Bayesian threshold is *t*().

**Figure 1:**
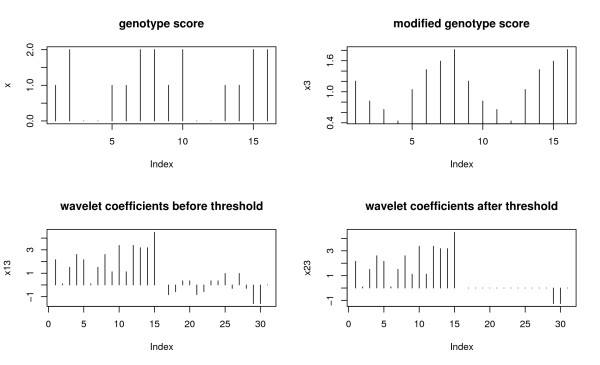
**Before and after wavelet transform**. The process of transforming original genotype data to its wavelet coefficients, thresholding the wavelet coefficients, and obtaining the modified genotype data by inverse transform of the thresholded wavelet coefficients.

After thresholding wavelet coefficients of genotype scores, we apply inverse wavelet transformation to obtain modified genotype scores. A score test is formed based on those modified genotype scores. The three step procedure of wavelet transformation is illustrated in Figure 1, where a genotype score of an individual is transformed into wavelet coefficients, which are then thresholded and transformed back to modified genotype scores.

### Wavelet-based score test

Consider a sample of *n *independent individuals genotyped at *m *SNPs, and let *X *= (*X*_*ij*_) be a matrix of genotypes, where *X*_*ij *_is the coded genotype of the *i*th individual at the *j*th SNP according to additive, recessive, or dominant disease models. For a given window, SNPs are recoded to maximize the number of positive pairwise correlation among them. For an additive model, at each SNP, one homozygous genotype is coded as 0, the other homozygous genotype is coded as 2, and the heterozygous genotype is coded as 1. This yields a sample genotype matrix. If at some SNPs, the coding of two homozygous genotypes is interchanged, a different sample genotype matrix will be obtained. Among all possible different sample genotype matrices, we choose the one that has the maximum number of positive entries in its sample variance-covariance matrix. This is done individually for each set of SNPs. This coding is always unique when applied to real data and it removes any ambiguity in the coding of genotypes. In our simulation studies, this recoding process did not inflate the type I error rate. Let *Y *= (*Y*_*i*_) be the trait value vector, where *Y*_*i *_is the trait value of the *i*th individual. The values *Y*_*i *_can represent qualitative or quantitative traits. For a case-control design, *Y*_*i *_= 1 for affected individuals and *Y*_*i *_= 0 for unaffected individuals. We assume the trait values and genotypes are related by a generalized linear model [16]



where *f *is the link function and *X*_*i *_= (*X*_*i*1_, *X*_*i*2_,..., *X*_*im*_). We first find the wavelet coefficients of *X*_*i*_. We then threshold the wavelet coefficients using empirical Bayesian thresholding. Finally, we apply inverse wavelet transform to the thresholded wavelet coefficients to obtain the modified genotype scores, *x*_*ij*_. This is done for every individual. If two individuals have the same genotypes, then they will have the same modified genotypes as well. A score statistic is formed as follows. Let



The sample variance of *U*_*j *_is



The score test statistic is defined as



The empirical *p*-values are calculated by permutations. The permutation approach may have limitations for GWAS. However, when we applied this method to a data of about 2,000 individuals with 500,000 SNPs, it took less than a week to run on a cluster with 23 nodes and 65 CPUs, which is still manageable.

### Simulations

For a case-control design, we compare the proposed score test based on wavelet transform (*T*_*w*_) with the following commonly used tests: the score test based on Fourier transform (*T*_*f*_); a test obtained by fitting a regression function with one SNP, followed by Bonferroni correction to find the global *p*-value (*T*_*b*_); and a likelihood-ratio test based on logistic regression (*T*_*l*_). For quantitative traits, we compare *T*_*w *_with *T*_*f*_, *T*_*b*_, and PCReg [8] (*T*_*p*_). The reasons for choosing these tests to be compared with the proposed test are the following: we chose *T*_*f *_because both *T*_*w *_and *T*_*f *_are affected by the order of the SNPs, but we want to show that *T*_*w *_adapts better to sparse and dense data than *T*_*f*_; we chose *T*_*b *_because it is a common and very effective test when one SNP has a strong association with the disease. If several SNPs have small to moderate information, a regression with multiple SNPs may have advantages, which is the reason behind our choice of *T*_*l*_. Both *T*_*b *_and *T*_*l *_are not affected by the order of SNPs, and they do not suppress data to reduce the degrees of freedom. The strength of *T*_*p *_lies on data suppression. A comparison of *T*_*w *_with *T*_*f*_, *T*_*b*_, *T*_*l*_, and *T*_*p *_demonstrated that *T*_*w *_achieves a higher power by effectively suppressing noise and by using extra information regarding the ordering of the SNPs.

#### Case-control design

The type I error rates and the powers of *T*_*w*_, *T*_*f*_, *T*_*b*_, and *T*_*l *_were analyzed. In our simulation study, there are 200 cases and 200 controls, and the number of SNPs is *m *+ 1 with the SNP at the center being an unobserved disease SNP. The allele frequencies of *m *non-disease SNPs are obtained from a uniform distribution between 0.2 and 0.8, and the allele frequency of the disease SNP is *p*. The haplotypes of the *m *+ 1 SNPs are generated from a multivariate normal distribution with a variance-covariance matrix (*ρ*_*ij*_). The *m *+ 1 allele frequencies give the cutoff points, which translate a multivariate normal vector to a haplotype. The sum of two haplotypes is a genotype vector. We can only observe the genotype data of cases and controls on *m *SNPs; the genotype of the disease SNP is not observable.

**Figure 2:**
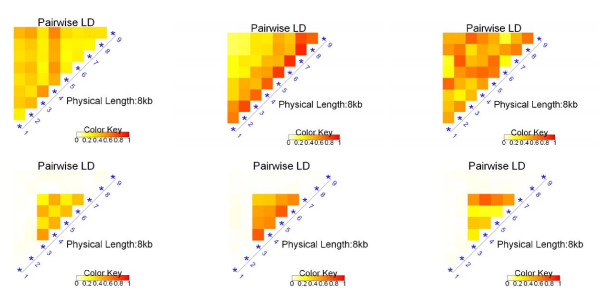
**LD patterns**. Pairwise *D' *for models with six different LD structures.

One of the objectives of our simulation studies was to test the adaptability of the proposed test. To this end, we considered six LD structure models. In the first three models, all SNPs were associated with the unobserved disease locus and they were also associated with each other in various ways. In the other three models, half of the SNPs were related to neither the disease locus nor to each other, and the other half of the SNPs were related to the disease locus and also to each other in ways similar to the first three models. Variance-covariance matrices of the multivariate normal distribution were used to generate different LD structures. We used six matrices A1-A6. For *i *≠ *j*, matrices A1 is given by *ρ*_*ij *_= 0.4; A2 is given by *ρ*_*ij *_= 0.8^|*i*-*j*|^; A3 is given by *ρ*_*ij *_~ Unif(0.3, 0.7); A4 is given by *ρ*_*ij *_= 0.4 if 1 +  ≤ *i*, *j *≤ 1 + , and *ρ*_*ij *_= 0 otherwise; A5 is given by *ρ*_*ij *_= 0.8^|*i*-*j*| ^if 1 +  ≤ *i*, *j *≤ 1 + , and *ρ*_*ij *_= 0 otherwise; A6 is given by *ρ*_*ij *_~ Unif(0.3, 0.7) if 1 +  ≤ *i*, *j *≤ 1 + , and *ρ*_*ij *_= 0 otherwise. Matrices A4-A6 represent situations where the first and last quarters of SNPs are related to neither the disease nor other SNPs, and they are purely noise. To demonstrate the LD structures in these six scenarios, we generated a simulated sample population of size 10,000 for each scenario, and calculated pairwise *D'*. The results are shown in Figure 2. The trait value was generated from the genotype of the disease SNP according to a multiplicative model and a relative risk (*RR*). To check the type I error rate (when RR = 1) and power, we let *RR *= 1, 1.15, 1.25, 1.5, 2, 2.5, *m *= 8 and the disease allele frequency *p *= 0.4. For each of the six models, the simulation was repeated 1,000 times, and the proportion with a *p *value less than 0.05 was recorded.

#### HapMap data

Next we applied the wavelet-based test to three genes: CHI3L2, IL21R, and CTLA4, which have also been analyzed in other simulation studies [8,10,17]. We downloaded genotype data of 60 individuals (parents) from CEPH (Utah residents with ancestry from northern and western Europe) in the HapMap [18]. In the following selections, we only chose SNPs with missing genotypes less than 5%.

In a 20 kb region around CHI3L2, we chose SNPs with minor allele frequencies greater than 0.26. This leads to 17 SNPs (rs755467, rs2255089, rs2494004, rs1325284, rs2251715, rs961364, rs2251608, rs2764543, rs2477574, rs2477578, rs942694, rs942693, rs2182114, rs5003369, rs3934922, rs3934923, and rs8535). During simulation studies, we randomly picked one SNP as the disease locus, and assumed it was not observed. We sampled a genotype at the disease locus from 60 individuals, and assigned its trait value (0 or 1) according to its genotype using a multiplicative disease model. A sample of 200 cases and 200 controls was then obtained. Next we generated genotypes of each case and control at the other 16 SNPs by sampling from individuals with the same genotype at the disease locus. Suppose we only observed genotype data at these 16 loci. If the disease SNP was among the observed SNPs, single locus methods should have higher power than multilocus methods. The proposed test is not intended to replace single locus methods under this situation. We only claim that the new test has a higher power when none of the SNPs in the window has a strong association with the disease by itself, in which case single locus method may fail to detect signals. In a region of 20 kb around IL21R, we chose SNPs with minor allele frequencies greater than 0.25. This yields 21 SNPs, in which we chose the largest block containing nine SNPs (rs179766, rs7203086, rs2040790, rs11074861, rs7199138, rs8057551, rs8061992, rs9930086, and rs8049804). A similar data set as before was generated from these nine SNPs.

In a region of 200 kb around CTLA4, there are 84 SNPs with minor allele frequencies greater than 0.25. These 84 SNPs cover eight blocks. Some of the SNPs are highly correlated (correlation is 1 for several SNPs). We took nine tagging SNPs chosen by Haploview [19], which captured 78 of the 84 alleles with a mean *r*^2 ^of 0.943 (rs2882969, rs11571293, rs1427680, rs231727, rs6705593, rs17268364, rs4355090, rs10183087, and rs3096747). A data set was generated from these nine SNPs in a similar fashion as before. Power comparisons were also done for 12 sites on chromosomes 1-10, 17, and 22, which have been studied before [17]. The genotype data of 60 parents from CEPH population in the HapMap [18] were used to generate samples as described before. We chose nine SNPs at each site with minor allele frequencies greater than 0.2. A SNP is randomly selected as the causal locus which is removed from the sample, and it varies during the simulation. The observed data consists of genotypes of 200 cases and 200 controls at eight SNPs. The locations of the 12 sites are given in Table 1.

**Table 1:** Positions of 12 sites on 12 chromosomes.

**Positions**			
**Chromosome 1**	**Chromosome 2**	**Chromosome 3**	**Chromosome 4**

76037376-76046029	165039214-165049745	48194541-48262883	95708105-95724998

Chromosome 5	Chromosome 6	Chromosome 7	Chromosome 8

86318185-86336013	85438224-85448287	79276265-79290697	73096328 -73104287

Chromosome 9	Chromosome 10	Chromosome 17	Chromosome 22

120732024-120745981	67665462-67693578	40283580-40300822	33626551-33661562

#### Quantitative traits

The new test can also be applied to quantitative traits. We compared the power and the type I error rates of *T*_*w *_with three commonly used tests *T*_*f*_, *T*_*b*_, and *T*_*p*_. The model used to generate trait values is *y *= *μ*(*x*_1 _+ *βx*_2_) + *e*, where *e *is a standard normal random variable; *x*_1 _= 1, 0, -1 and *x*_2 _= 0, 1, 0 for genotypes *DD, Dd, dd*, respectively; and *β *= -1, 0, 1 for recessive, additive, and dominant models, respectively. We calculated *μ *from the heritability, which ranges from 4% to 10%.

PCReg [8] is described as follows. Let *G *be the matrix of genotypes, and let *y *be the vector of trait values. Suppose *y *and the columns of *G *are all centered such that their means are zeros. Let the columns of *A *be the first several characteristic vectors of *G*^*T*^*G *such that they can explain more than 80% of the total variation in *G*. Let *G*_1 _= *GA *be the projections of the genotype data onto these principal directions. The regression model is *y *= *G*_1_*b*_1 _+ ϵ, and *T*_*p *_is the regression *F *-statistic. We used permutation to calculate the empirical *p*-value of *T*_*p*_.

## Results

In Table 2, the results show that the wavelet-based test *T*_*w *_has the correct type I error rates.

**Figure 3:**
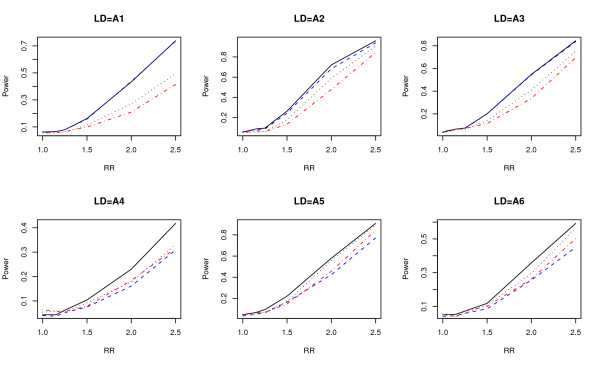
**Power comparisons using simulated data**. Power comparisons of the wavelet-based test (*T*_*w*_), the Fourier-based test (*T*_*f*_), the single locus test with Bonferroni correction (*T*_*b*_), and the likelihood-ratio test based on logistic regression (*T*_*l*_) with eight SNPs for a case-control design. The black solid, blue dashed, black dotted, and red dot-dash lines are *T*_*w*_, *T*_*f*_, *T*_*b*_, and *T*_*l*_, respectively.

Power comparisons under the six LD structures described before are given in Figure 3. From Figure 3 we can see that *T*_*w *_and *T*_*f *_have similar powers in models 1-3, and they both have a higher power than *T*_*b *_and *T*_*l*_. For models 4-6, *T*_*w *_still has the highest power among the four tests, but *T*_*f *_is not as good as the other two tests. The wavelet-based test always has the highest power regardless of whether the window of SNPs contains SNPs unrelated to the disease. The wavelet-based test *T*_*w *_is robust with respect to the noise level (SNPs unrelated to the disease). When all SNPs in a window are related to the disease, the wavelet-based test does not suppress noise too much to lose power, and when some of the SNPs are unrelated to the disease, it automatically suppress noise more to reduce the number of degrees of freedom and to keep its power.

**Table 2:** Type I error rates for tests with qualitative traits.

**Type I error rates**						
**LD structures**	**LD = A1**	**LD = A2**	**LD = A3**	**LD = A4**	**LD = A5**	**LD = A6**

*T*_*w*_	0.061	0.056	0.039	0.043	0.046	0.050

*T*_*f*_	0.063	0.059	0.037	0.040	0.035	0.041

*T*_*b*_	0.055	0.050	0.036	0.054	0.052	0.049

*T*_*l*_	0.056	0.049	0.046	0.063	0.053	0.060

The window size is eight. Assuming multiplicative disease model. *T*_*w *_is the wavelet based test, *T*_*f *_is the Fourier based test, *T*_*b *_is the single SNP test with Bonferroni correction, and *T*_*p *_is the principal component regression test.

**Figure 4:**
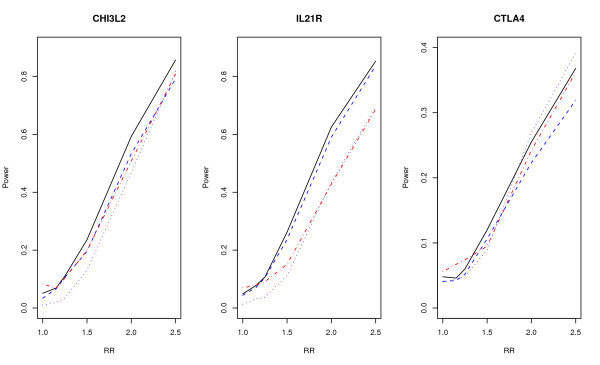
**Power comparisons using HapMap data 1**. Data sampled from CHI3L2, IL21R, and CTLA4 on HapMap. Power comparisons of the wavelet-based test (*T*_*w*_), the Fourier-based test (*T*_*f*_), the single locus test with Bonferroni correction (*Tb*), and the likelihood-ratio test based on logistic regression (*T*_*l*_) for case-control design. The black solid, blue dashed, black dotted, and red dot-dash lines are *T*_*w*_, *T*_*f*_, *T*_*b*_, and *T*_*l*_, respectively.

Simulation results for data generated from the HapMap data on CHI3L2, IL21R, and CTLA4 are given in Figure 4. In all cases, the score test based on wavelet clearly has the highest power. For the other three tests, there is no clear winner. For the 17 SNPs around CHI3L2, the proposed test has the highest power. The score test based on Fourier transform is the second when the relative risk is 1.5 or 2, while the single locus test with Bonferroni correction is the second when the relative risk is 2.5. For the nine SNPs on a block around IL21R, the proposed test and the test based on Fourier transform have similar powers, although the power of the new test is slightly higher. The powers of the other two tests are clearly lower. For the nine tagging SNPs covering eight blocks around CTLA4, the power of the new test is again the highest under all situations. This shows that the proposed test has a higher power under various LD structures.

Note that in the above simulations, the causal locus is randomly chosen among all SNPs around a gene. Therefore, it is free to vary during simulations. Second, the causal locus is not observed in any simulations because the intent of the proposed test is to provide a tool to detect small to moderate associations in a window of SNPs when none of them have a strong LD with the disease. We expect that if the causal locus is observed, the power of single locus tests will increase more than those of multilocus tests. Third, the window sizes in our simulations are eight and 16. When the window size is eight, we can recode genotypes to obtain as many positive pairwise correlations as possible. For larger window sizes it will not be feasible because of computational burden. When genotypes are recoded, it is done for all individuals regardless of their phenotypes.

**Figure 5:**
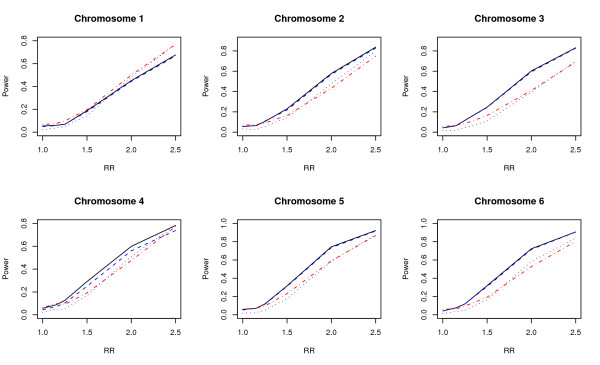
**Power comparisons using HapMap data 2**. Data sampled from six sites on chromosomes 1-6 on HapMap. Power comparisons of the wavelet-based test (*T*_*w*_), the Fourier-based test (*T*_*f*_), the single locus test with Bonferroni correction (*T*_*b*_), and the likelihood-ratio test based on logistic regression (*T*_*l*_) with eight SNPs for case-control design. The black solid, blue dashed, black dotted, and red dot-dash lines are *T*_*w*_, *T*_*f*_, *T*_*b*_, and *T*_*l*_, respectively.

**Figure 6:**
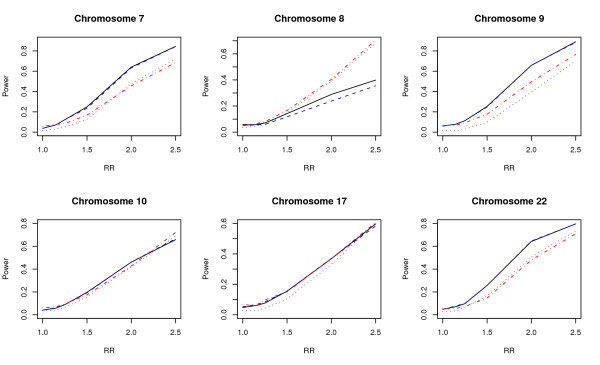
**Power comparisons using HapMap data 3**. Data sampled from six sites on chromosomes 7-10, 17, and 22 on HapMap. Power comparisons of thewavelet-based test (*T*_*w*_), the Fourier-based test (*T*_*f*_), the single locus test with Bonferroni correction (*T*_*b*_), and the likelihood-ratio test based on logistic regression (*T*_*l*_) with eight SNPs for case-control design. The black solid, blue dashed, black dotted, and red dot-dash lines are *T*_*w*_, *T*_*f*_, *T*_*b*_, and *T*_*l*_, respectively.

The comparison of powers on 12 sites on chromosomes 1-10, 17, 22 are given in Figures 5 and 6 which clearly demonstrate the superiority in power for the proposed test.

**Figure 7:**
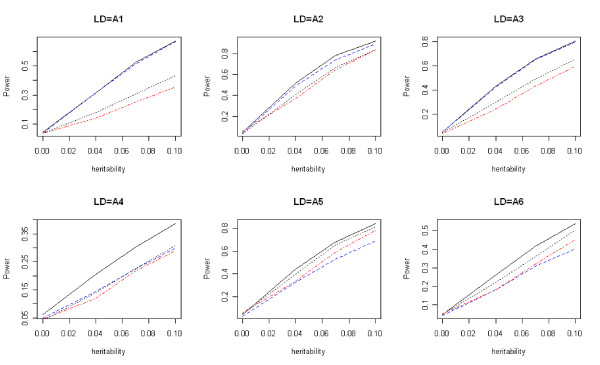
**Power comparisons for quantitative traits (dominant)**. Power comparisons of the wavelet-based test (*T*_*w*_), the Fourier-based test (*T*_*f*_), the single locus test with Bonferroni correction (*T*_*b*_), and PCReg (*T*_*p*_) with eight SNPs for quantitative traits. The black solid, blue dashed, black dotted, and red dot-dash lines are *T*_*w*_, *T*_*f*_, *T*_*b*_, and *T*_*p*_, respectively. Assume the dominant disease model.

**Figure 8:**
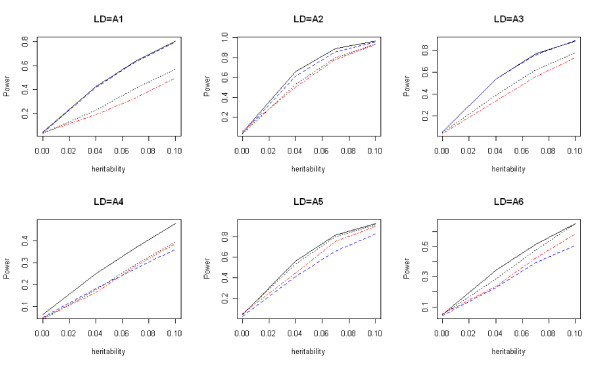
**Power comparisons for quantitative traits (additive)**. Power comparisons of the wavelet-based test (*T*_*w*_), the Fourier-based test (*T*_*f*_), the single locus test with Bonferroni correction (*T*_*b*_), and PCReg (*T*_*p*_) with eight SNPs for quantitative traits. The black solid, blue dashed, black dotted, and red dot-dash lines are *T*_*w*_, *T*_*f*_, *T*_*b*_, and *T*_*p*_, respectively. Assume the additive disease model.

**Figure 9:**
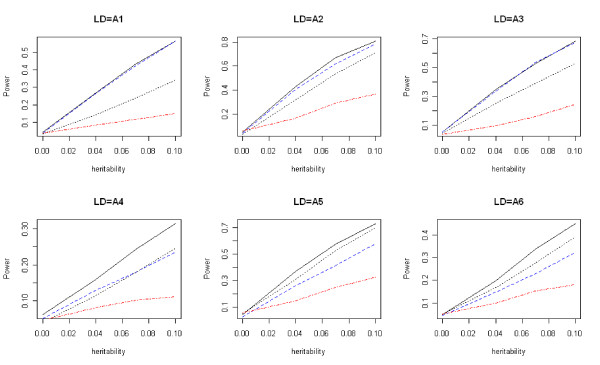
**Power comparisons for quantitative traits (recessive)**. Power comparisons of the wavelet-based test (*T*_*w*_), the Fourier-based test (*T*_*f*_), the single locus test with Bonferroni correction (*T*_*b*_), and PCReg (*T*_*p*_) with eight SNPs for quantitative traits. The black solid, blue dashed, black dotted, and red dot-dash lines are *T*_*w*_, *T*_*f*_, *T*_*b*_, and *T*_*p*_, respectively. Assume the recessive disease model.

For quantitative traits, the type I error rates of *T*_*w*_, *T*_*f*_, *T*_*b*_, and *T*_*p *_are given in Table 3. Comparisons of the power of these four tests are given in Figures 7, 8, and 9. The results are similar to those for the qualitative traits, which shows that *T*_*w *_has the highest power and correct type I error rate.

**Table 3:** Type I error rates for tests with quantitative traits.

**Type I error rates**						
**LD structures**	**LD = A1**	**LD = A2**	**LD = A3**	**LD = A4**	**LD = A5**	**LD = A6**

*T*_*w*_	0.044	0.049	0.050	0.061	0.040	0.047

*T*_*f*_	0.039	0.040	0.052	0.050	0.025	0.042

*T*_*b*_	0.036	0.033	0.045	0.041	0.050	0.050

*T*_*p*_	0.040	0.060	0.037	0.045	0.055	0.051

The window size is eight. Assuming additive disease model. *T*_*w *_is the wavelet based test, *T*_*f *_is the Fourier based test, *T*_*b *_is the single SNP test with Bonferroni correction, and *T*_*p *_is the principal component regression test.

## Discussion

Simulation studies show that the proposed test achieves a higher power than other commonly used tests. The improved power results from three sources. The first is the use of the wavelet transformation of genotype data. The wavelet transform is designed to deal with unsmooth signals with jumps and small wiggles. Genetic data are not smooth nor periodic, which is naturally dealt with by the wavelet transform. Using the transformed data instead of the original data enables us to view the signals in different frequencies and in different resolution levels separately. The second is the choice of thresholds in the wavelet transformation. The wavelet transformation decomposes data into coefficients corresponding to different frequencies and to different resolution levels. It is generally believed that a low frequency signal is more likely to be a true signal than a high frequency one, and a true signal is more sparse on a fine resolution level than on a coarser level. Suppressing wavelet coefficients at different frequencies and different resolution levels in various ways increases the effectiveness of the noise suppression, which means that the data can be represented by using fewer wavelet coefficients. Empirical Bayesian thresholding automatically decides how much noise to be suppressed at each level according to the data. A wavelet transform with empirical Bayesian thresholding gives the proposed test its ability to adapt to LD structures: it suppresses more if many SNPs under consideration are unrelated with the disease, and it suppresses less if most SNPs are in fact associated with the disease.

The third reason for the improvement of the power comes from taking the relative positions of SNPs on a chromosome into consideration. An important difference between wavelet-based tests and PCReg is that PCReg does not consider the relative positions of SNPs. It views a multilocus genotype as a vector. The wavelet-based test treats a multilocus genotype as a discretized function instead of a vector. The difference between regarding genotypes as a function versus a vector is that viewing it as a function allows us to take the order of SNPs on a chromosome into consideration. The importance of the ordering of SNPs on a chromosome can be illustrated by the following simple example. Suppose in a GWAS, one finds two locations. At one location, multilocus genotype 1#1#1# appears frequently among cases; and at another location ###111 appears often among cases, where # represents noise and 1 represents a heterozygous genotype. The question is which location is more likely to be a true signal, and which one is more likely to be noise. For PCReg, the two locations have the same importance. Projecting onto the first, the third, and the fifth dimensions is the same as projecting onto the fourth, the fifth, and the sixth dimensions. However, for a wavelet-based test, they are different. A wavelet-based test considers multilocus genotypes as discretized functions, ###111 represents a low frequency function, while 1#1#1# represents a high frequency function. If everything else remains the same, a low frequency function is more likely to be a true signal, and a high frequency function is more likely to be noise. It is worth noticing that for tests based on haplotype sharing, ###111 is also more important than 1#1#1# because the former will increase shared length of haplotypes. In the Appendix, we prove that many genotype-based tests and some haplotype-based tests do not utilize the information contained in the spatial order of SNPs.

## Conclusion

We propose a score test based on a wavelet transformation. The goal is to increase power by suppressing noise and therefore reducing the number of degrees of freedom. The adaptability of the empirical Bayesian thresholding provides the test with the ability to automatically suppress the right amount of noise which is shown by simulation studies using HapMap data. Whether the window contains SNPs related to the disease or not, the proposed test always has the highest power comparing with the single marker test with Bonferroni correction and the likelihood-ratio test based on logistic regression. This shows the effectiveness of the noise suppression by the proposed test. The second advantage of the proposed test is that it takes the order of SNPs on a chromosome into consideration. In this sense, it is a compromise between genotype-based tests and haplotype-based tests. Since it considers the order of SNPs, it uses more information than other genotype-based methods, while avoiding the need to infer unobserved haplotypes or their frequencies as in haplotype-based tests. Simulation studies show that the proposed test consistently has a higher power than PCReg. The proposed test and PCReg both suppress data to reduce the number of degrees of freedom to increase the power. The major difference between them is that the proposed test takes the order of SNPs into consideration while PCReg does not. The difference between their powers show that considering the order of SNPs does increase the power of the tests. The proposed test and the test based on the Fourier transform have similar powers when all SNPs in a window are related to the disease and the wavelet-based test has a higher power when some of the SNPs are not related to the disease. This demonstrates the advantage of using the wavelet transform than using the Fourier transform. Since genotype data are not smooth nor periodic, it is naturally better dealt with by the wavelet transform. Although the proposed test has many advantages, it is certainly not universally better than other tests. For example, if a window contains a SNP strongly associated with the disease, a single locus method with Bonferroni correction should be better than any multilocus methods, including the new test. In a GWAS, researchers usually apply single locus methods first, and report significant findings if there are any. Only after that initial step, multilocus methods are used to identify information missed by the single locus methods. The proposed test should be used with this in mind.

If population stratification is a concern, we suggest to apply EIGENSTRAT [20] to the data to obtain several large principal components. These principal components are used to adjust genotypes and phenotypes as suggested by Price et al. [20]. The wavelet-based test is calculated using adjusted genotypes and phenotypes. The genomic inflation factor *λ*_*GC *_[21] can be used as a criterion to determine if population structures are present. The new test has been successfully applied to a GWAS of the North American Rheumatoid Arthritis Consortium data from Genetic Analysis Workshop 16 [22].

## Web Resource

wavelet-based score test, 

## Authors' contributions

RJ and JD both contributed in the development of the statistical test, provided simulation strategies, and drafted the manuscript. RJ also participated and guided the numerical calculations. YD carried out part of the programing work. All authors read and approved the manuscript.

## Appendix

In the appendix we prove that *T*_*p*_, the Hotelling's *T*^2 ^test, *T*_*b*_, and *T*_*l *_are not affected by permuting SNPs. Let *G *= (*g*_*ij*_) be an *n *× *m *genotype matrix, where *g*_*ij *_is the genotype of the *i*th individual at the *j*th marker. Subtract the mean of the *j*th column from *g*_*ij *_such that the mean of each column of *G *is 0. The sample covariance matrix of the genotypes is *A *= *G*^*T*^*G*/(*n *- 1). Suppose that *λ*_1 _≥ *λ*_2_≥...≥*λ*_*m *_are the eigenvalues of *A*, and *v*_1_, *v*_2_,..., *v*_*m *_are the corresponding eigenvectors. Let *D *be a diagonal matrix with *λ*_1 _≥ *λ*_2_≥...≥*λ*_*m *_as diagonal entries, and let *V *be an *m *× *m *matrix with *v*_1_, *v*_2_,..., *v*_*m *_as columns. Then *A *= *VDV*^-1^. Note that *v*_*i *_is the *i*th principal component, *i *= 1, 2,..., *m*. Write *V *= [*V*_1_*V*_2_], where *V*_1 _contains the used principal components, and *V*_2 _contains the discarded principal components. In PCReg, the regression model is *y *= *GV*_1_*b *+ ϵ.

Suppose the spatial order of SNPs is permuted, then the columns of *G *are also permuted accordingly. Suppose the new genotype matrix is , then  = *GP *where *P *is obtained from applying the same permutation on the columns of the identity matrix. Assume that  is also centered so that the mean of each column is 0. Let . Since *P *is an orthogonal matrix, *P*^*T *^= *P*^-1^. Therefore,  = *P*^*T*^*G*^*T*^*GP *= *P*^*T*^*VDV*^-1^*P *= (*P*^*T*^*V*)*D*(*P*^*T*^*V*)^-1^. Note that the diagonal matrix of the eigenvalues of  is still *D*, and the matrix of eigenvectors of  is *P*^*T*^*V*. Since *A *and  have the same eigenvalues, the used principal components are the columns of  and the discarded principal components are the columns of . In PCReg, the regression model after permutation is  which is the same as before permuting SNPs.

Next, we prove that the Hotelling's *T *^2 ^statistic is not affected by permuting SNPs. Following the notations used in [3], let *X *be an *n*_1 _× *m *matrix of genotypes of cases and let *Y *be an *n*_2 _× *m *matrix of genotypes of controls, where *n*_1 _is the number of cases, *n*_2 _is the number of controls, and *m *is the number of SNPs. Let  and  be the column mean of *X *and *Y*, respectively, written as column vectors. Let *X*_*i *_and *Y*_*i *_denote the *i*th row of *X *and *Y*, respectively, written as column vectors. The pooled-sample variance-covariance matrix of genotypes is



The Hotelling's *T*^2 ^statistic is



Write , where **e**_**i **_is the *i*th column of the identity matrix, and **1 **is a column vector with every entry being 1. Thus,



where *I *is the identity matrix and *E *is a square matrix with every entry being 1. Therefore,



Suppose the spatial order of SNPs are permuted, the corresponding columns of the genotype matrices *X *and *Y *are permuted accordingly. After permutation, let  and  be the genotype matrix of cases and controls, respectively. Then  = *XP *and  = *YP*, where *P *is a permutation matrix. The new pooled-sample variance-covariance matrix is



After permutation,  and  becomes  and , respectively. Recall that *P*^-1 ^= *P*^*T*^. The Hotelling's *T*^2 ^statistic after permuting the spatial order of SNPs is



The same arguments can be applied to prove that the haplotype *T*^2 ^statistic defined in [3] is not affected by permuting SNPs either. It was proved in [3] that both the multilocus *T*^2 ^and the haplotype *T*^2 ^statistics have the same power. Usually a haplotype-based test will have a higher, or at least a different, power than a genotype-based test. It is a interesting fact that both the multilocus *T*^2 ^and the haplotype *T*^2 ^have the same power and neither have used the information contained in the spatial order of SNPs.

Recall that *T*_*b *_is obtained by fitting a regression function with one SNP, followed by Bonferroni correction to find the global *p*-value. Permuting SNPs does not change its results.

The likelihood-ratio test based on logistic regression *T*_*l *_is also not affected by permuting SNPs.
